# Supplementary material for: Considerations on the Kinetic Processes in the Preparation of Ternary Co-Amorphous Systems by Milling
Source: Pharmaceutics. 2023 Jan 3;15(1):172. doi: 10.3390/pharmaceutics15010172 (PMC9866880; doi:10.3390/pharmaceutics15010172)
Supplement: Supplementary file 1 [file pharmaceutics-15-00172-s001.zip › pharmaceutics-2021454-supplementary.pdf]

pharmaceutics

MDPI

Supplementary Materials: Considerations on the Kinetic Processes in the Preparation of Ternary Co-Amorphous Systems by Milling

Yixuan Wang, Thomas Rades, and Holger Grohganz

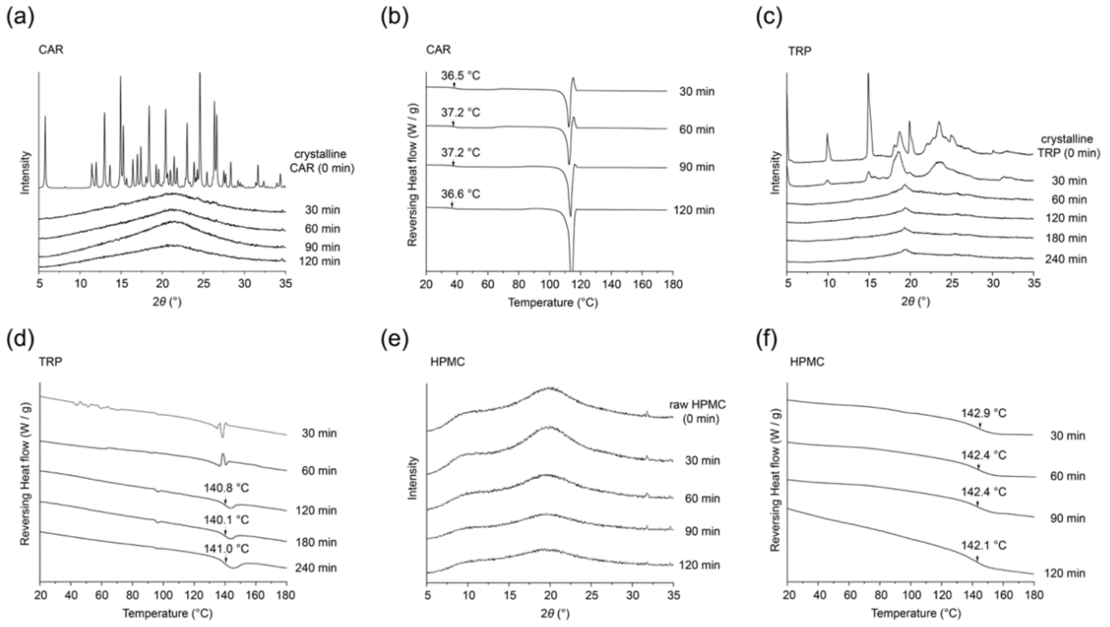

Figure S1. XRPD diffractograms and DSC thermograms for CAR (a, b), TRP (c,d) and HPMC (e,f) after ball milling. The T_gs are indicated by arrows.

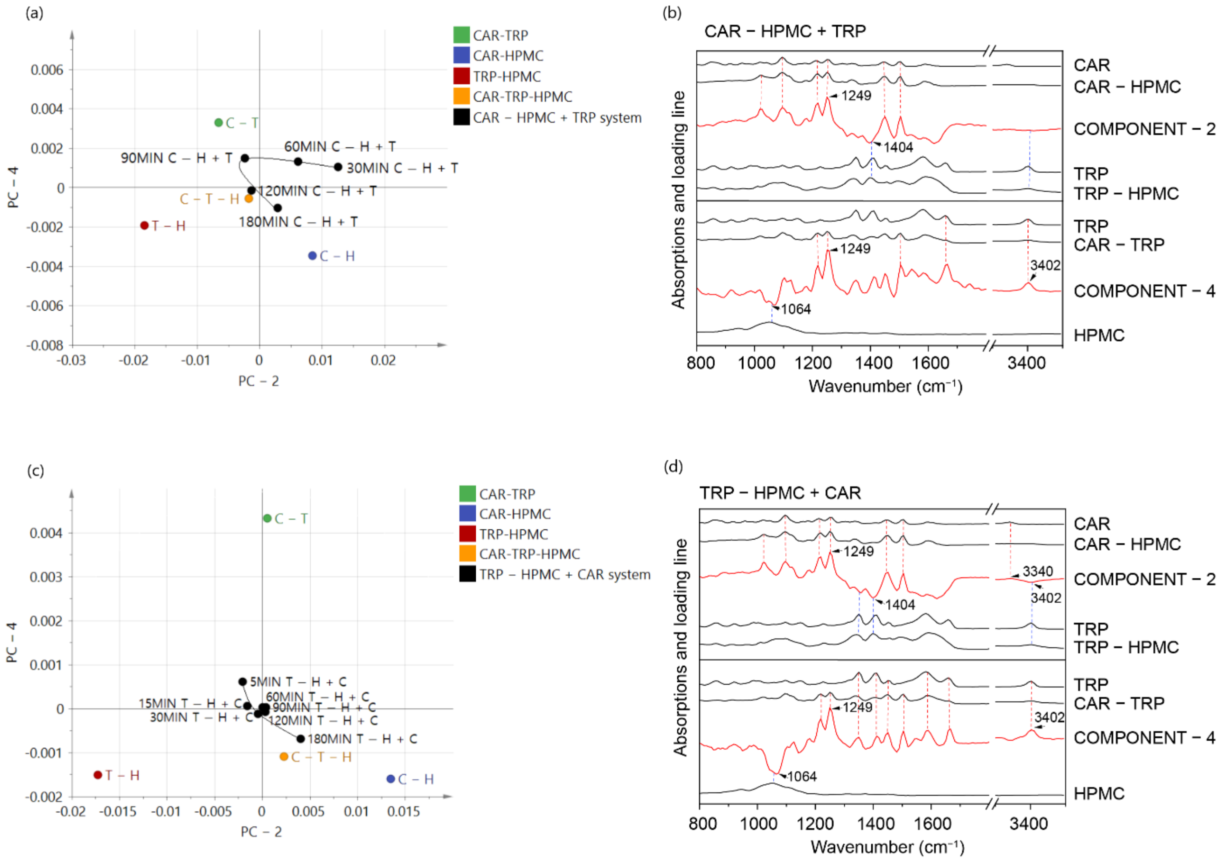

Figure S2. : Score and loading plots of the PCA on FT-IR data for CAR – HPMC + TRP and TRP – HPMC + CAR. Score plot of the PCA on FT-IR data (a) , the loading plot of PC – 2 and PC – 4 (b) of the CAR – HPMC + TRP co-amorphous system;. Score plot of the PCA on FT-IR data (c) , the loading plot of PC – 2 and PC – 4 (d) of the TRP – HPMC + CAR co-amorphous system.

1. PCA of CAR-HPMC+TRP ternary system

In PC – 2, positive loading values corresponded to the FT-IR spectra of the CAR in CAR – HPMC, while negative loading values matched well with the FT-IR spectra of TRP in TRP – HPMC; In PC – 4, positive loading values corresponded to the FT-IR spectra of the TRP in CAR – TRP, while negative loading values matched with the FT-IR spectra of HPMC (Figure S2b).

In the score plot of the PCA, the CAR – HPMC + TRP amorphous sample shows a leftward trend from 30 min to 90 min and a right-down trend from 90 min to 180 min (Figure S2a). According to the PC loadings, the interaction of TRP with the other components in the system became more prominent for the first 90 min, and the prevalence of CAR – HPMC decreased. After 90 minutes, the portion of CAR – HPMC increased, and CAR – TRP decreased. This development is consistent with the T_g data (Figure 2d).

2. PCA of TRP – HPMC + CAR ternary system

In PC – 2, positive loading values corresponded to the FT-IR spectra of the CAR in CAR – HPMC, while negative loading values matched well with the FT-IR spectra of the TRP in TRP – HPMC; In PC-4, positive loading values corresponded to the FT-IR spectra of the TRP, while negative loading values matched with the FT-IR spectra of HPMC (Figure S2d).

In the score plot of the PCA, the TRP – HPMC + CAR A – B + C amorphous sample shows a right-down trend with increasing ball milling ball milling times (Figure S2c), corresponding in an increase in CAR – HPMC which is consistent with the T_g data reflected by DSC (Figure 2f).
